# Laparoscopic surgical treatment for hydrocele of canal of Nuck: A case report and literature review

**DOI:** 10.1186/s40792-021-01205-8

**Published:** 2021-05-13

**Authors:** Liming Wang, Taku Maejima, Susumu Fukahori, Katayose Shun, Daitaro Yoshikawa, Toru Kono

**Affiliations:** Department of Surgery, Sapporo Higashi Tokushukai Hospital, 3-1, N-33, E-14, Higahi-ku, Sapporo, Hokkaido 0650033 Japan

**Keywords:** Hydrocele of canal of Nuck, Laparoscopy, Anterior approach

## Abstract

**Background:**

Hydrocele of canal of Nuck (HCN) is a rare disease in adult female. The diagnosis and treatment of HCN is still a challenge for surgeons.

**Case presentation:**

A 56-year-old female presented with recent onset of occasional pain during exercise and an asymptomatic left groin swelling. Ultrasonography results were suspicious for left inguinal hernia incarceration and computed tomography (CT) scan showed no intestinal obstruction, which was considered as HCN. Laparoscopic hydrocelectomy of the HCN and a routine laparoscopic hernia repair via the transabdominal preperitoneal (TAPP) approach were performed. Postoperative pathology showed no malignant lesions or endometriosis.

**Conclusions:**

The preoperative diagnosis of HCN is extremely important. Surgeons should choose appropriate surgical methods for different anatomical HCNs based on the preoperative diagnosis.

## Background

Hydrocele of canal of Nuck (HCN) is a very rare disease in adult female [1], most of which manifests as swelling of the groin. It is often accidentally misdiagnosed as an inguinal hernia or an incarcerated inguinal hernia which requires emergency surgery [2]. The standard treatment is to completely remove HCN through open anterior surgery [3, 4]. However, how to accurate diagnose and treat HCN has become a common problem for traditional treatment methods [5]. This paper describes a case of HCN that was repaired laparoscopically. We review the literature and discuss the strategy of the surgical treatment for HCN.

## Case presentation

**Fig. 1:**
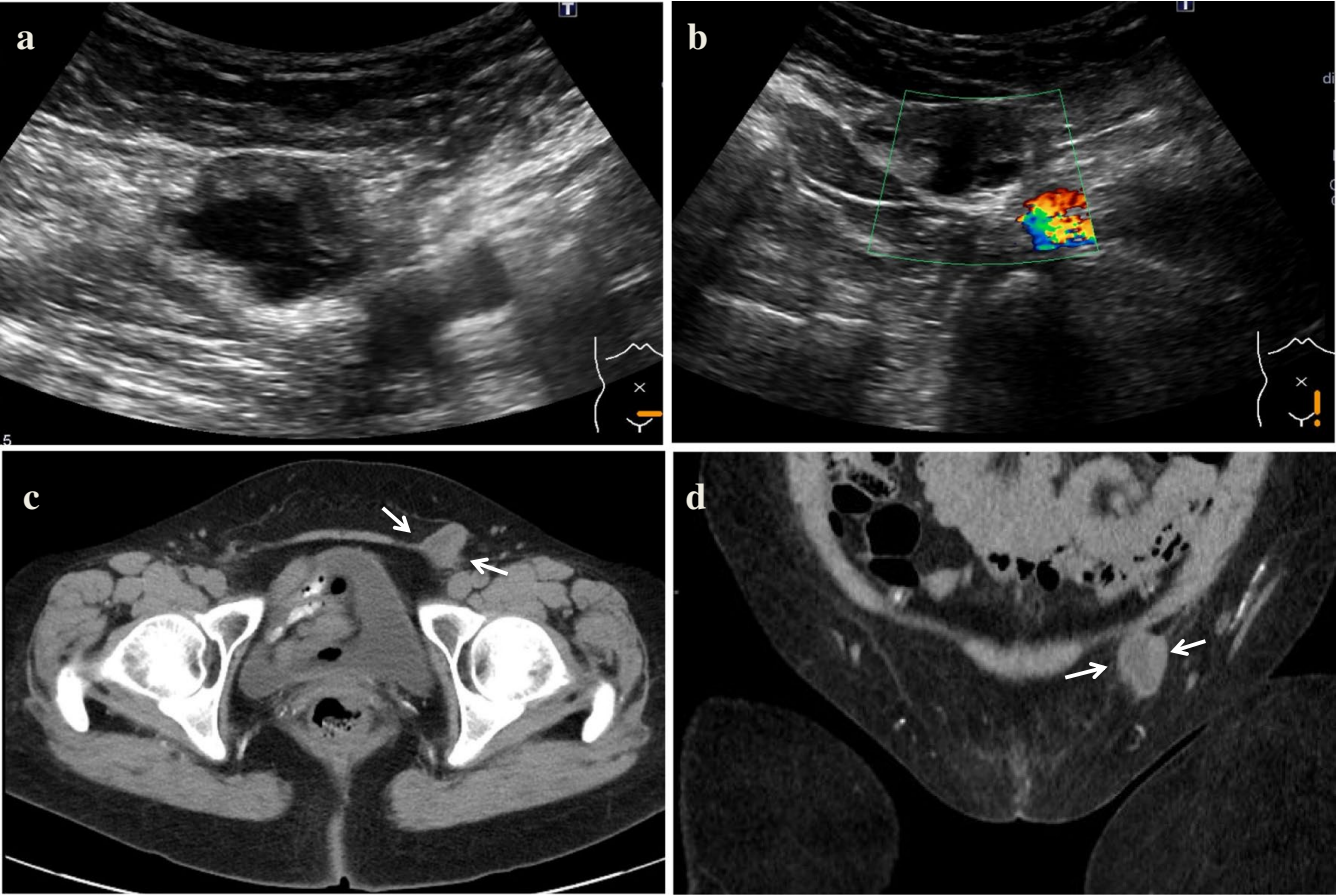
Ultrasound images **a** US showed a cystic lesion measuring 40 mm in diameter, which had unclear boundaries and appeared hypoechoic in the inguinal canal. **b** The color Doppler scan did not show any internal blood vessel distribution. **c** CT showed the inside of the cystic lesion was liquid, with irregular hypertrophy of the cyst wall. **d** CT coronal image shows that the cystic is not communicating with the abdominal cavity

A 56-year-old female patient on dialysis presented with swelling of the left inguinal area which was aggravated during activities and suspected to be an inguinal hernia. Palpation revealed that the left groin mass could not be returned to the abdominal cavity. Ultrasound results of the lower groin (US) was suspect for intestinal obstruction (Fig. 1a, b). However, computed tomography (CT) scan showed that the cystic lesion did not communicate with the abdominal cavity and there was no significant enlargement compared with imaging performed three years ago (Fig. 1c, d); this was preliminarily diagnosed as HCN.

**Fig. 2:**
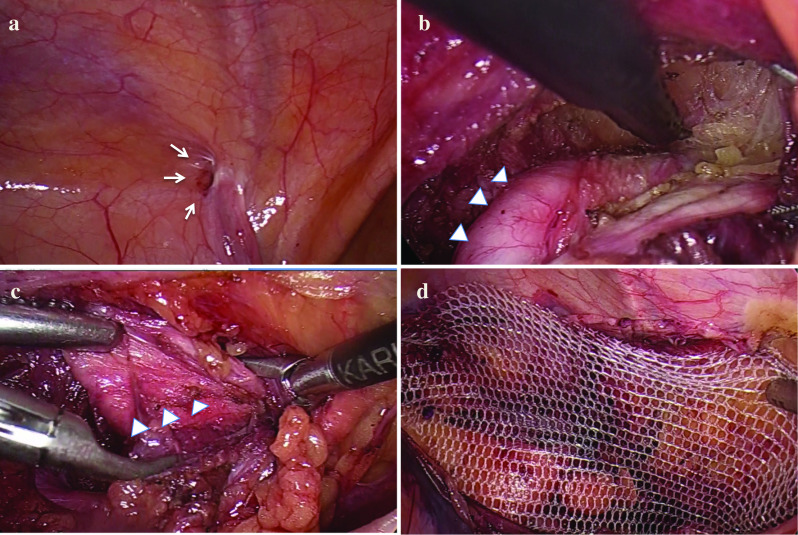
Intraoperative photos. **a** The internal inguinal ring is weak, but no obvious hernia sac (white arrows). **b** Type B HCN, completely located in the inguinal canal (white arrowheads). **c** The round ligament was cut off and the posterior wall of HCN was dissociated from the cephalic side until the HCN was finally removed completely. **d** A lightweight polypropylene mesh was introduced to cover the inguinal myopectineal triangle and absorbable tacks were used to fix the mesh

Under general anesthesia, a 12-mm trocar was inserted through the umbilical incision, and two 5-mm working ports were placed symmetrically. The internal inguinal ring was relatively weak (Fig. 2a), but there was no obvious hernia sac. After carefully dissociating the peritoneal cavity and pulling on the round ligament, we could see the HCN located entirely within the inguinal canal close to the skin (Fig. 2b). Since the inferior epigastric vessels were at risk of injury, we added a 5-mm trocar in the left lower abdomen so that the assistant could pull the round ligament of uterus. Because the cyst was hard, it was difficult to perform further dissection at the distal end of hydrocele from the ventral cavity side. Therefore, we cut off the round ligament and freed the posterior wall of the hydrocele from the cephalic side until the hydrocele was eventually completely removed (Fig. 2c). The specimen was wrapped in an endo-bag and taken out through the 12-mm trocar. After confirming that there was no bleeding in the inguinal canal, a lightweight polypropylene mesh was introduced to cover the inguinal myopectineal triangle and absorbable tacks were used to fix the mesh (Fig. 2d). The operative time was about two hours.

**Fig. 3:**
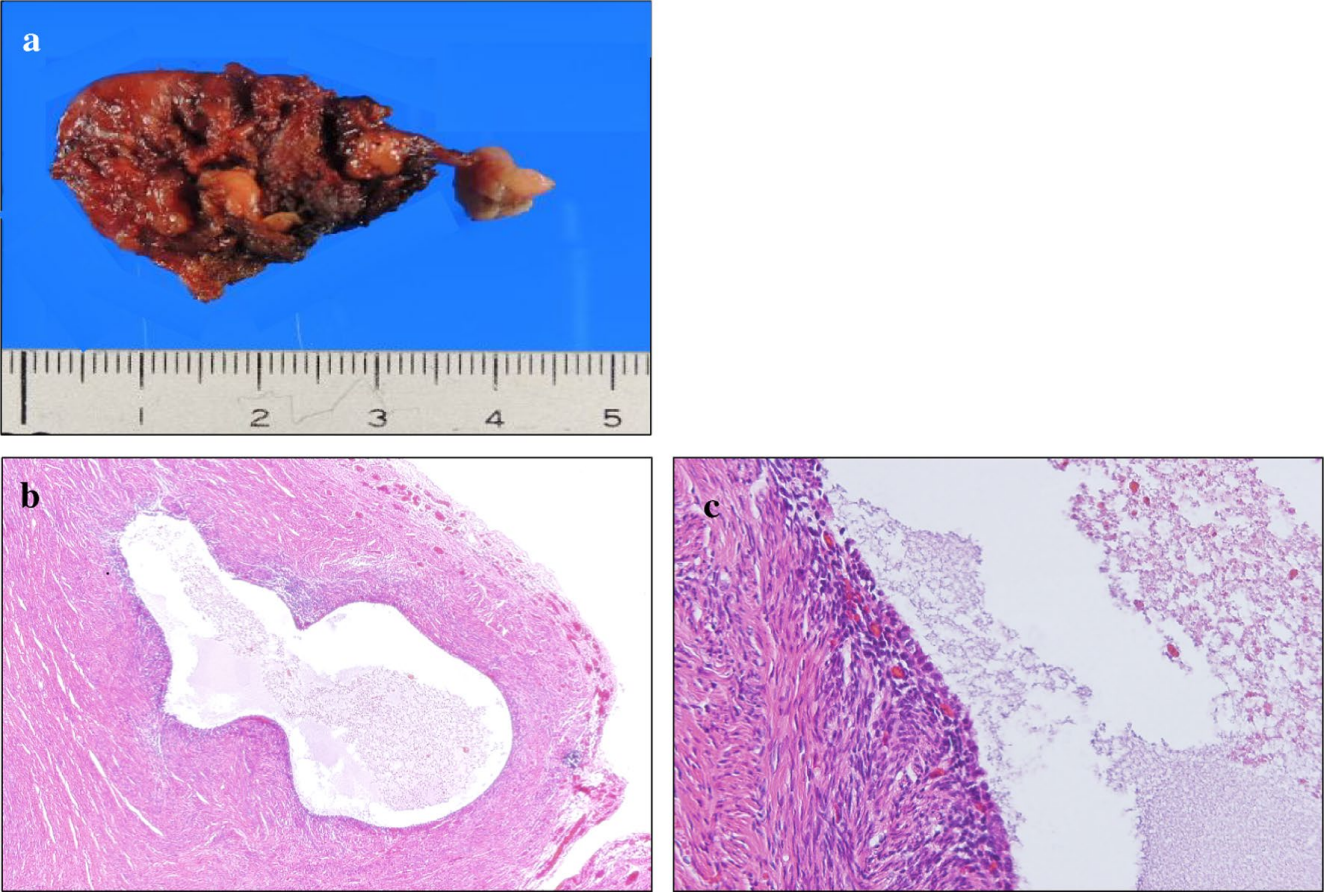
Resected specimen and pathological results. **a** A macroscopic examination showed that the resected specimen was a 4 × 3 cm, solid mass with no obvious effusion. **b** The hydrocele consisted of muscle tissue, fibrous tissue, a small amount of adipose tissue, and dilated blood vessels with hyperemia (hematoxylin and eosin [HE], × 40). **c** A lining tubular structure can be seen on the cubic epithelium lacking aberrations (HE, × 100)

The patient recovered well after the operation with a non-invasive infection and was discharged on post-operative day one. The resected specimen was a 4 × 3 cm solid mass with no obvious effusion (Fig. 3a). Histological examination showed that the hydrocele consisted of muscle, fibrous tissue, a small amount of adipose tissue, and dilated blood vessels with hyperemia. A lining tubular structure can be seen on the cubic epithelium lacking aberrations; no endometrial glands and no malignancy was observed (Fig. 3b, c).

## Discussion

**Fig. 4:**
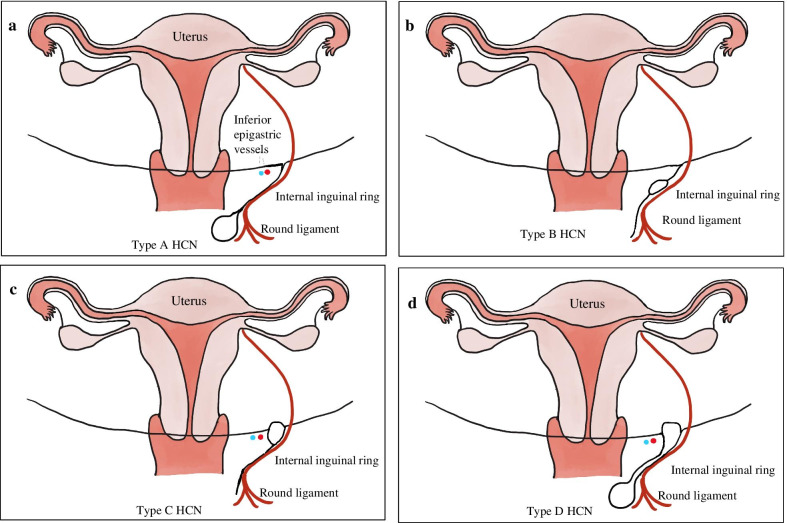
HCN classifications. **a** Type A: HCN is located subcutaneously over the inguinal canal. **b** Type B: HCN is located in the inguinal canal. **c**Type C: HCN is limited to the internal inguinal ring. **d** Type D: HCN spreads from the internal inguinal ring to the inguinal canal or subcutaneously

In 1691, the anatomist from the Netherlands Anton Nuck first described the canal of Nuck in a female [6], which was similar to hydrocele of the spermatic cord in males. Failure to occlude can result in an inguinal hernia [7, 8], incomplete occlusion could result in fluid retention in the peritoneum, which was known as HCN. HCNs descend along the round ligament of uterus and are classified into communicative and non-communicating cysts according to the presence or absence of traffic in the abdominal cavity [1]. Communicative cysts are more common in infants under one year of age [9]. So HCN in adult female was rare. In this paper, in order to facilitate intraoperative understanding, we divided HCNs into four types according to the anatomical position: Type A, HCN is located subcutaneously over the inguinal canal (Fig. 4a); Type B, HCN is located in the inguinal canal (Fig. 4b); Type C, HCN is limited to the internal inguinal ring (Fig. 4c) and Type D, HCN spreads from the internal inguinal ring to the inguinal canal or subcutaneously (Fig. 4d). Our case belonged to Type B, although internal inguinal ring was relatively weak, there was no internal inguinal ring defect. Completely in laparoscopic surgery, the internal inguinal ring must be repaired with a patch. This increased the time and cost of the operation and was worthy of our rethinking.

The manifestations of HCN were described as lumps in the groin area, with varying degrees of pain, size, and reducibility. Therefore, it was usually misdiagnosed as an inguinal hernia [10, 11]. Although US can be used as a convenient method of examination [12], CT scan or magnetic resonance imaging provide much more accurate images [13], including the anatomical relationship between cystic lesions and adjacent structures, to confirm whether it communicates with the peritoneal cavity. Even so, when we do not see a hernia sac that matches the preoperative examination during laparoscopic hernia repair, we need to pay attention to whether female patients may have HCN [4, 14].

According to previous reports, about a third of HCNs have been reported to be associated with inguinal hernias [9, 15]. As the application of laparoscopy in hernia repair has matured in recent years, laparoscopy can not only be used as a diagnostic method to determine whether there is a complicated hernia or tumor, but also as a treatment method to remove the HCN or tumor and perform an inguinal triangle patch repair [10, 16].

**Fig. 5:**
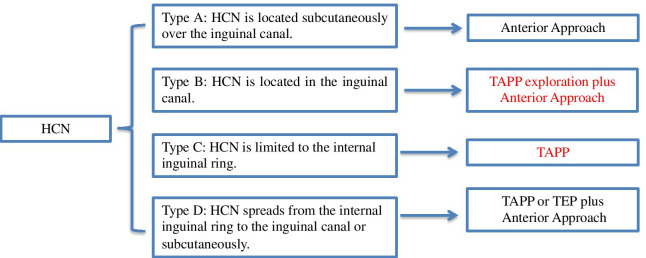
The strategy of the surgical treatment for HCN

However, laparoscopy also has limitations compared to anterior approach surgery. Since the inferior epigastric artery may interfere with the surgical field of vision [16, 17], it is necessary to adjust the surgical trocar or open the posterior wall of the inguinal canal [6]. In addition, it has been reported that HCN may be associated with endometriosis [3, 18] or benign tumors and malignancy [9, 19, 20]. Therefore, ensuring the complete resection of HCN is particularly important. If laparoscopic resection is difficult, the courage to convert to the traditional anterior approach is required [18, 21] (Fig. 5).

Laparoscopic TAPP resection and TEP (totally extraperitoneal) have become progressively more popular methods [10, 22]. However, compared with TEP, TAPP allows better imaging of the lesions in the abdominal cavity [2, 10], although it may cause intraperitoneal adhesions [22]. As for the choice of TEP or TAPP, it is actually determined by the surgeon's proficiency. If the preoperative diagnosis is not too accurate, TAPP may provide more diagnostic information [14] (Table 1) (Fig. 5).

**Table 1.** The advantages and disadvantages of various surgical approaches

Approach	Anterior approach	TAPP	TEP	TAPP plus anterior approach
Advantages	Simple and convenient, short operation time; prevents intraperitoneal tumor spreading	Accurate diagnosis, especially when intraperitoneal organs or tumors are incarcerated in inguinal hernia	Prevents adhesions in the abdominal cavity. Easier to separate HCN from the inguinal canal than TAPP	Accurate intraoperative diagnosis and quick resection of HCN; selectively treated for various HCNs
Disadvantages	Intraperitoneal organs incarcerated in the inguinal canal are not easy to handle	Inferior epigastric vessels interfere with surgical field of vision; a patch may be needed	Intraperitoneal observation is not possible; a patch would be needed	A patch would be needed if HCN removed by TAPP

Even though laparoscopy has so many advantages, it is difficult for the laparoscope to successfully free the distal end of the HCN if the inguinal canal is deep or the HCN is too huge [21]. At this point, once laparoscopy finds that the patient has pure HCN, it is recommended to immediately perform anterior resection to shorten the operation time.

## Conclusion

The preoperative diagnosis of HCN is extremely important. Surgeons should choose appropriate surgical methods for different anatomical HCNs based on the preoperative diagnosis.

## Data Availability

The datasets supporting the conclusions of this article are included within the article and its additional files.

## Abbreviations

HCN: Hydrocele of canal of Nuck
CT: Computed tomography
US: Ultrasonography
TEP: Totally extraperitoneal
TAPP: Transabdominal preperitoneal

## References

[1] Counseller VS, Black BM (1941). Hydrocele of the canal of Nuck: report of seventeen cases. Ann Surg.

[2] Shahid F (2020). Laparoscopic hydrocelectomy of the canal of Nuck in adult female: case report and literature review. Int J Surg Case Rep.

[3] Uno Y (2014). Mesothelial cyst with endometriosis mimicking a Nuck cyst. J Surg Case Rep.

[4] Kim KS (2016). Hydrocele of the canal of Nuck in a female adult. Arch Plast Surg.

[5] Qureshi NJ, Lakshman K (2014). Laparoscopic excision of cyst of canal of Nuck. J Minim Access Surg.

[6] Chihara N (2020). Use of a novel open posterior wall technique for laparoscopic excision of hydrocele of the canal of Nuck in an adult female: case report. J Nippon Med Sch.

[7] Yen CF (2001). Laparoscopic closure of patent canal of Nuck for female indirect inguinal hernia. J Am Assoc Gynecol Laparosc.

[8] Topal U (2018). Cyst of the canal of Nuck mimicking inguinal hernia. Int J Surg Case Rep.

[9] Nasser H (2018). Anatomy and pathology of the canal of Nuck. Clin Imaging.

[10] Russell JC (2001). Laparoscopic closure of patent canal of Nuck for female indirect inguinal hernia. J Am Assoc Gynecol Laparosc.

[11] Cheng, E.M., A. Sarkar, and D.S. Perera, *Hydrocoele in the canal of Nuck in an adult female: a rare cause for inguinal swelling.* ANZ J Surg, 2020. 10.1111/ans.16279 32840952

[12] Khanna PC (2007). Sonographic appearance of canal of Nuck hydrocele. Pediatr Radiol.

[13] Chan D (2019). Canal of Nuck hernias. Acta Radiol Open.

[14] Bunting D, Szczebiot L, Cota A (2013). Laparoscopic hernia repair–when is a hernia not a hernia?. JSLS.

[15] Fikatas P (2020). Hydroceles of the canal of Nuck in adults-diagnostic, treatment and results of a rare condition in females. J Clin Med.

[16] Kojima S, Sakamoto T (2020). Laparoscopic total extraperitoneal treatment for a hydrocele of the canal of Nuck located entirely within the inguinal canal: a case report. Asian J Endosc Surg.

[17] Kohata, A., et al., *Large hydrocele of the canal of Nuck diagnosed and treated using conventional and laparoscopic methods.* J Surg Case Rep, 2020. **2020**(8): rjaa222. 10.1093/jscr/rjaa222 PMC7446026 32864093

[18] Swatesutipun, V., et al., *Endometriosis in the Canal of Nuck presenting with suprapubic pain: A case report and literature review.* Urol Case Rep, 2021. **34**: 101497. 10.1016/j.eucr.2020.101497 PMC7691153 33294379

[19] Josefsson ML, Mitra S, Gupta S (2013). Inguinal ovary in adult women-case report and literature review. Springerplus.

[20] Motooka Y (2018). Radical resection of an endometrioid carcinoma arising from endometriosis in the round ligament within the right canal of Nuck: a case report and literature review. Gynecol Oncol Rep.

[21] Wang L (2021). Laparoscopic assisted hydrocelectomy of the canal of Nuck: a case report. Surg Case Rep.

[22] Matsumoto T (2014). Laparoscopic diagnosis and treatment of a hydrocele of the canal of Nuck extending in the retroperitoneal space: a case report. Int J Surg Case Rep.

